# Publisher Correction: ESICM LIVES 2023

**DOI:** 10.1186/s40635-023-00575-7

**Published:** 2023-12-18

**Publisher Correction: Intensive Care Medicine Experimental 2023, 11(Suppl 1):72** 10.1186/s40635-023-00546-y

Following publication of the original article [1], it came to the journal’s attention that due to a technical error, the abstract of the following details had been omitted from the supplement:

## 000714

### Starting with recognition: using education to improve CAM-ICU assessment in a District General Hospital’s Intensive Care Unit

#### M. Salamanca^1^, A. White^1^, C. Coyle^2^

##### ^1^Newham University Hospital, Barts Health NHS Trust, London, United Kingdom; ^2^Royal London Hospital, Barts Health NHS Trust, London, United Kingdom

**Correspondence**: M. Salamanca

This abstract has since been added to the supplement. The publisher thanks you for reading this erratum and apologizes for any inconvenience caused.
